# Space sequestration below ground in old-growth spruce-beech forests—signs for facilitation?

**DOI:** 10.3389/fpls.2013.00322

**Published:** 2013-08-20

**Authors:** Andreas Bolte, Friederike Kampf, Lutz Hilbrig

**Affiliations:** ^1^Thünen Institute of Forest EcosystemsEberswalde, Germany; ^2^Department of Silviculture and Forest Ecology of the Temperate Zones, University of GöttingenGöttingen, Germany

**Keywords:** *Fagus sylvatica*, *Picea abies*, root system stratification, fine root biomass (FRB), fine root length (FRL), fine root surface area index (RAI), specific root length (SRL), specific root surface area (SRA)

## Abstract

Scientists are currently debating the effects of mixing tree species for the complementary resource acquisition in forest ecosystems. In four unmanaged old-growth spruce-beech forests in strict nature reserves in southern Sweden and northern Germany we assessed forest structure and fine rooting profiles and traits (≤2 mm) by fine root sampling and the analysis of fine root morphology and biomass. These studies were conducted in selected tree groups with four different interspecific competition perspectives: (1) spruce as a central tree, (2) spruce as competitor, (3) beech as a central tree, and (4) beech as competitor. Mean values of life fine root attributes like biomass (*FRB*), length (*FRL*), and root area index (*RAI*) were significantly lower for spruce than for beech in mixed stands. Vertical profiles of fine root attributes adjusted to one unit of basal area (*BA*) exhibited partial root system stratification when central beech is growing with spruce competitors. In this constellation, beech was able to raise its specific root length (*SRL*) and therefore soil exploration efficiency in the subsoil, while increasing root biomass partitioning into deeper soil layers. According to relative values of fine root attributes (*rFRA*), asymmetric below-ground competition was observed favoring beech over spruce, in particular when central beech trees are admixed with spruce competitors. We conclude that beech fine rooting is facilitated in the presence of spruce by lowering competitive pressure compared to intraspecific competition whereas the competitive pressure for spruce is increased by beech admixture. Our findings underline the need of spatially differentiated approaches to assess interspecific competition below ground. Single-tree approaches and simulations of below-ground competition are required to focus rather on microsites populated by tree specimens as the basic spatial study area.

## Introduction

There is an on-going scientific debate about the effects of mixing tree species on forest ecosystem functioning in terms of productivity and resource acquisition. Most of the recent publications indicate a superiority of mixed-species stands compared to pure stands (Chen et al., 2003; Kelty, 2006; Erickson et al., 2009; Lei et al., 2009). For mixed forests with European beech (*Fagus sylvatica* L.) and Norway spruce [*Picea abies* (L.) Karst], Pretzsch and Schütze (2009) have presented a thorough analysis based on material from Southern Bavaria (Germany) presenting evidence for overyielding of the mixed stands above ground and growth acceleration of Norway spruce due to niche separation. These findings are supported by the previous review of Knoke et al. (2007) reporting a productivity increase compared to monospecific spruce and beech stands, but also higher stability against disturbances like storms, which was previously found by Schütz et al. (2006). All these considerations are mainly focused on species interaction and productivity aspects above ground, while root-system interactions play a minor role.

However, studies on fine root distribution in mixed forest with European beech (*Fagus sylvatica* L.) show evidence of species interaction and its effects on tree performance and stability. Several studies in managed mature beech-spruce mixtures in Germany and Austria found indications for vertical root system stratification (Rothe, 1997; Schmid, 2002; Bolte and Villanueva, 2006) supporting the idea of complementary resource acquisition of mixed spruce and beech stands both above- and below-ground. Lei et al. (2012a) also found a shift of fine root allocation in Norway spruce in the presence of other tree species studying fine root competition in a multispecies experiment with younger trees (BIOTREE, Germany). In contrast, publications from multispecies broadleaved forests with beech at Hainich National Park (Germany) found no evidence for root system stratification and overyielding below ground (Meinen et al., 2009a,b; Jacob et al., 2013). The different habit of rooting distribution found for beech in different tree species mixtures with either an indication of root system stratification (e.g., MacQueen, 1968; Büttner and Leuschner, 1994; Hendriks and Bianchi, 1995; Rust and Savill, 2000) or none (e.g., Curt and Prévosto, 2003; Meinen et al., 2009b) is explained by the variation in growth and space occupation dynamic of beech and its competitors which may be related to their different successional status (Bolte and Villanueva, 2006; Meinen et al., 2009a). Beech has been identified as being favored in interspecific competition below ground with less competitive tree species like Norway spruce [*Picea abies* (L.) Karst.] or oak (*Quercus* ssp., Büttner and Leuschner, 1994; Bolte and Villanueva, 2006; Rewald and Leuschner, 2009).

Due to climate change, mixtures of European beech with Norway spruce may attain new attraction in forestry in both the hemi-boreal zone and the montane-temperate zone, since beech is supposed to: (1) expand its distribution range northwards (Bradshaw and Lindbladh, 2005), (2) be more resistant than spruce to an increase in abiotic and biotic stress due to warming, drought, and insect attacks, and (3) gain on productivity compared to spruce (Bolte et al., 2010, in press; Grundmann et al., 2011). However, we lack knowledge on the root system structure and distribution in natural mixed spruce-beech forests to evaluate the coherence or difference of fine-rooting comparing temperate and hemiboreal spruce-beech forests. Moreover, more information is needed about spatial effects of interspecific competition and constellations of tree mixture on fine root distribution and structure, since almost all previous studies provided results only on a stand scale.

The present study was aimed at (1) finding evidence for vertical root system stratification in the unmanaged spruce-beech forests, (2) quantifying effects of spruce-beech competition in different mixture constellations on fine root structural traits, and (3) evaluating the competitive status of both species below ground considering different levels of interspecific competitive pressure.

Our present study includes a temperate near-natural forest within the high montane zone of the Harz Nationalpark (Northern Germany) with climate and site conditions comparable to the hemi-boreal forests of our other two study sites in Southern Sweden (see Table 1). We applied a sophisticated sampling design that allows us to differentiate spatial effects of interspecific competition and tree mixture constellations. The results will be discussed comparing our findings with recent knowledge from studies on spruce-beech interactions.

**Table 1:** **Location and site parameter of the three study sites**.

	**Rågetaåsen**	**Siggaboda**	**Rehberg**
Location	Southern Sweden, Halland	Southern Sweden, Småland	Northern Germany, Lower Saxony (Harz)
Geographic coordinates	56°51′ N 13°06′ E	56°27′ N 14°33′ E	51°43′ N 10°33′ E
Elavation (m a. sl.)	140–160	140–165	651–700
Exposure	SE	varying	SE
Mean annual temperture (°C)	6.4	6.0	6.1
Precipitation (mm a^−1^)	1200	700	1200
Humus type	Moder to raw humus	Raw humus	Moder to raw humus
Bedrock	Gneiss	Gneissic granite	“Grauwacke” sandstones and “Hornfels” (partly with Loess overlay)
Soil texture	Sandy silt	Silty sand	Sandy silt
Soil type	Podzolic Cambisol	Podzolic Cambisol	Podzolic Cambisol
Moisture-status	Good (partly boggy)	Good (partly boggy)	Good
Nutrition status	Poor to moderate	Poor to moderate	Moderate
Stand age (years)	Spruce and beech >130 (+ natural regeneration)	Beech up to 230, spruce up to 210 (+ natural regeneration)	Spruce and beech around 150 (+ natural regeneration)
Proportion of spruce/beech (%)	50/50	60/40	65/35

## Materials and methods

### Site and stand description

The two Swedish old-growth forests with Norway spruce [*Picea abies* (L.) Karst.] and European beech (*Fagus sylvatica* L.) are located in the boreo-nemoral zone (Sjörs, 1999) within the counties of Halland and Småland (Table 1). The Halandish site at “Rågetaåsen” (Halmstad district) lies at the western fringe of the southern Swedish highlands with cool and humid climate, high precipitation rates and countless peat bogs and lakes. The site “Siggaboda” is situated within the south-eastern part of the forest and lake district of Småland near to the county borders to Blekinge and Skåne. Precipitation is distinctively lower than at the luv side, however, bogs and lakes are frequent due to the generally cool climate and a climatic water surplus. Comparable climatic conditions can be found at the German montane spruce-beech forest “Rehberg” within the “Harz Nationalpark” (Lower Saxony).

At the Swedish site, the bedrock is formed mainly by metamorphic rocks (granitic gneiss) covered with moraine sediments whereas at the Harz site both metamorphic “Hornfels” and sedimentary “Grauwacke” sandstones prevail, partly covered by loess (eolic silt sediment). All sites have comparably low amounts of fine-textured soil and are riddled with boulders. Soil traits of all three sites are comparable with a moderate to thick accumulation of organic material 5–12 cm; the humus type is moder to raw humus (Rågetaåsen, Rehberg) or raw humus (Siggaboda) with a high C/N ratio. Fine soil material is dominated by silt (40–65%) and sand (18–52%); the clay proportion is relatively low (8–17%) and the texture can be classified as sandy silt or silty sand, respectively. The soil type is a Haplic Podzol (BGR, 2007) with a high moisture status. Beside several small fens at the Swedish sites, the soils are drained and quite acidic, occurring in the aluminum and iron buffer range at Siggaboda and Rehberg and the ion exchange buffer range at Rågetaåsen (Ulrich, 1983, Table 2). The amount of exchangeable base cations is limited; the higher base cation supply in the humus layer and “Ahe” horizon at Rehberg is likely due to repeated soil liming to compensate for anthropogenic acidic deposition (Table 2, Dammann and Guericke, 2002). Site moisture status is sufficient (partly boggy at the Swedish sites) and nutrient status poor to moderate at all sites.

**Table 2:** **Selected chemical soil properties of the three stands**.

	**Rågetaåsen**	**Siggaboda**	**Rehberg**
**HUMUS LAYER (Of/Oh)**			
Thickness (cm)	5	>10	9
pH (KCl)	3.3–3.9	3.0–3.6	4.7–5.2
C/N ratio	23.8–23.9	28.9–31.0	22.5–27.6
**MINERAL SOIL (Ahe HORIZON)**			
Depth (cm)	0–5	0–10	0–8
pH (KCl)	4.4	3.4	4.4
CEC (μ mol_c_ g^−1^)	94.9	75.1	51.1
Al + Fe (% CEC)	58.9	64.8	38.2
K + Ca + Mg (% CEC)	29.9	30.0	58.8
**MINERAL SOIL (Bhs HORIZON)**			
Depth (cm)	5–10	10–16	8–24
pH (KCl)	4.5	3.6	3.7
CEC (μ mol_c_ g^−1^)	55.3	66.6	127.5
Al + Fe (% CEC)	75.0	81.9	76.7
K + Ca + Mg (% CEC)	16.3	13.2	22.8
**MINERAL SOIL (Bv−Cv HORIZON)**			
Depth (cm)	10–50+	16–45+	24–46+
pH (KCl)	4.8	4.7	4.0
CEC (μ mol_c_ g^−1^)	18.5	54.2	84.5
Al + Fe (% CEC)	77.5	83.1	85.9
K + Ca + Mg (% CEC)	15.9	12.8	13.0

*The ranges between minimum and maximum values of n combined samples consisting of at least 4 single samples taken at every subplot are displayed. Of, incompletely decomposed organic layer; Oh, humified organic layer containing amorphous organic material; Ahe, humified and eluviated mineral top soil horizon; Bhs, humified and podsolic mineral soil horizon; Bv–Cv, cambic mineral soil horizon; CEC, effective cation exchange capacity; Al + Fe, exchangeable aluminum and iron ions; K + Ca + Mg, sum of exchangeable base cations*.

All three mixed stands are dominated by spruce with higher mean values of stem density, diameter at breast height (*dbh*), tree heights, and basal area (BA) (Table 3). The Swedish stands are denser with higher *BA*s, in particular of spruce, whereas the spruce and beech trees at the German site Rehberg feature larger dimensions (both *dbh* and height).

**Table 3:** **Traits of the stand structure for spruce and beech of all mixed stand plots (*n* = 6 plots per stand)**.

**Stand**	**Rågetaåsen**	**Siggaboda**	**Rehberg**
**STEM DENSITY (TREE NUMBER per ha)**			
Spruce	370 ± *343*	310 ± *169*	55 ± *41*
Beech	148 ± *61*	179 ± *170*	43 ± *31*
Total	518 ± *264*	489 ± *175*	98 ± *35*
**MEAN *dbh* (cm)**			
Spruce	30.1 ± *14.3*	32.3 ± *13.3*	59.8 ± *6.8*
Beech	28.4 ± *9.3*	24.6 ± *14.4*	46.7 ± *10.4*
Total	29.3 ± *11.5*	28.1 ± *13.8*	52.7 ± *11.0*
**MEAN TREE HEIGHT (m)**			
Spruce	21.7 ± *7.1*	23.7 ± *5.6*	33.1 ± *2.1*
Beech	16.2 ± *6.8*	16.8 ± *4.1*	26.6 ± *3.5*
Total	18.9 ± *7.2*	19.9 ± *5.8*	29.6 ± *4.4*
**MEAN BASAL AREA (m^2^ ha^−1^)**			
Spruce	17.7 ± *12.4*	34.6 ± *28.1*	14.8 ± *10.5*
Beech	10.1 ± *6.4*	7.3 ± *5.9*	8.3 ± *6.4*
Total	27.8 ± *10.3*	41.9 ± *23.1*	23.1 ± *8.9*

*Mean values ± standard deviations are displayed*.

#### Field sampling

Forest stand structures were recorded in a 1 ha square core plot (100 × 100 m) in the center of the semi-natural forest representing a typical section of the old-growth stand. We subdivided the plot using a 20 m-grid (cf. procedure for German forest nature reserves, Meyer et al., 2001). In total 36 grid points of the core plot were leveled with an ultrasonic hypsometer (Vertex III, Haglöf Inc. Sweden) and a compass (PM-5/400PC, Suunto Inc., Finland) and subsequently semi-permanently marked with wooden stakes.

Within the entire 1 ha core plot, we assessed each tree with a *dbh* (tree diameter at 1.3 m above the ground) of 7 cm and larger in winter 2004 at Siggaboda and in spring 2005 at the other sites. For each tree, species, cardinal location coordinates of the stem using above mentioned equipment and dbh with a girth tape were recorded. Subsequently, a tagging system (Signumat, Latschenbacher, Austria) was used for temporarily (between 2004 and 2007) marking and numbering each measured tree.

**Figure 1:**
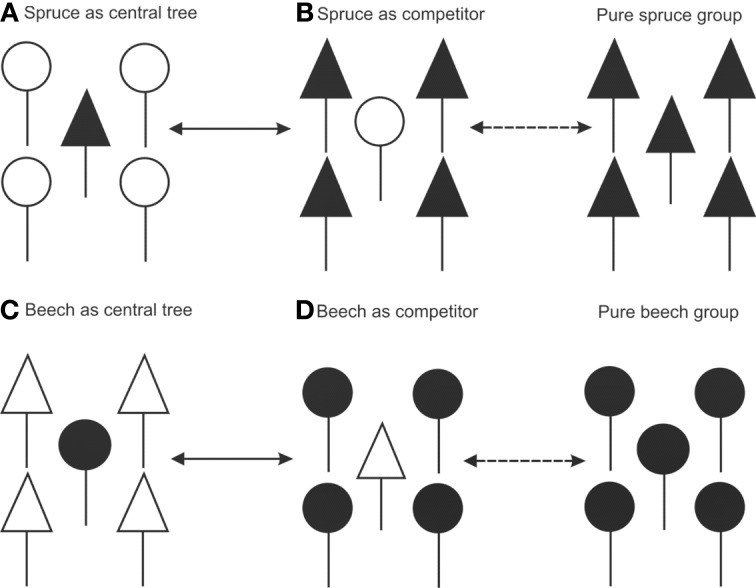
**Sampling design of tree groups with varying competitive status of mixed Norway spruce and European beech, and comparison with monospecies groups (only for analyses of mixed species representation)**.

**Figure 2:**
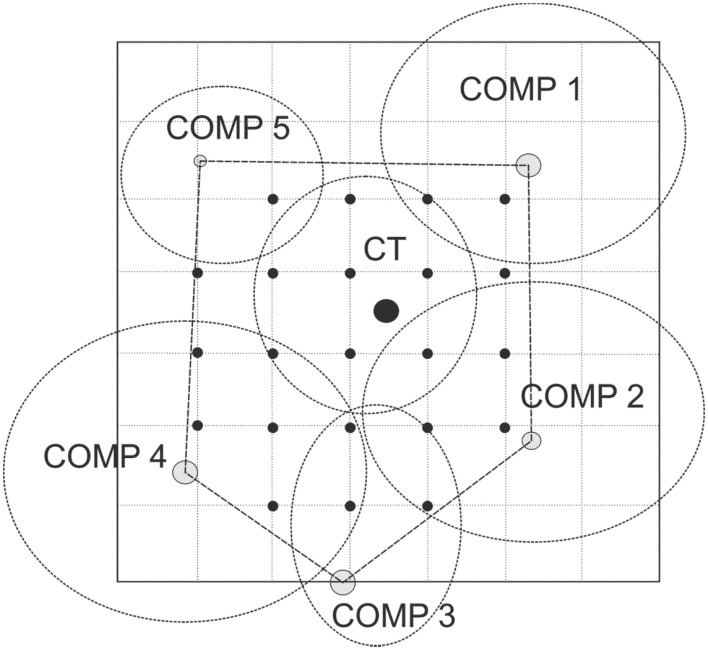
**Grid design for root sampling; *CT*, central tree; *COMP*, competitor. Black dots showing the positions of the soil core sampling**.

We studied fine root structure of mature trees focusing on tree groups with one central tree with its mostly four–five competitors (mean value 4.84). Therefore, beech-spruce tree groups representing four different types of interspecific competitive situations were selected: (**A**) spruce as the central tree, (**B**) spruce as competitors, (**C**) beech as the central tree, and (**D**) beech as competitors. This design was completed by monospecies groups with comparable intraspecific competitive status (Figure 1). All directly neighboring trees with crown interaction were defined as competitors. Fine root sampling was conducted in a 8 × 8-point grid of variable width from 1.12 to 5.36 m (Figure 2). Total sample area was determined by the distance between the central tree and the farthest competitor (i.e., half size of the quadrat's edge, see Figure 2), and ranged from 61 to 1408 m^2^. With this approach it was possible to assess the rooting system of the central tree with different extension but a comparable root sample number. Root sample number was reduced by excluding grid points outside the central tree—competitors range defined as a polygon area within competitors stem position (Figure 2). The resulting sample area was then 21 m^2^ to 402 m^2^. This interspecific sampling design was repeated three times at each of the three sites whereas we sampled only two intraspecific group constellations per plot (beech, spruce) that was only used as a reference for analyses of mixed species representation (see following chapter on data analyses).

At each grid point soil cores with a surface area of 20 cm^2^ were taken using a soil auger (d ~ 5 cm) until the solid bedrock layer was reached. The maximum mineral soil depth sampled varied from 20 to about 40 cm. Various studies revealed that the maximum of fine roots is found in the top soil layers (estimated >80%, cf. Büttner and Leuschner, 1994; Rothe, 1997; Hertel, 1999). Where the exact grid point position coincided with a solid boulder we moved the sample point within the grid orientation until fine soil was reached. In a few cases when large boulders covered several grid points the central sample points were skipped. All variations from the regular grid design were recorded with the exact position of the moved sample points.

#### Processing of root samples

All soil core samples including soil or humus material and roots were soaked in water overnight. We separated root substance from soil using the floating method described by Böhm (1979). The watered samples were filled into trays and roots floating on top of the water were sieved off using a 1-mm mesh. We repeated this procedure until only stones were left in the soil sample. Root material was extracted from the fine soil organic matter of the humus samples by gentle washing. Roots and the remaining soil organic matter floating dispersed in the water-filled trays were separated manually. Washed root samples were kept in de-ionized water at 4°C until sorting.

We classified all root parts less than or equal to 2 mm in diameter as fine roots and separated them from coarse roots which were not investigated further. Fine root sorting addressed species (spruce, beech, other tree and shrub/herb species) and vitality (live, dead). We applied morphological criteria for the identification of dead root material: dead root parts exhibits a dark discoloration of the central cylinder and a decreased flexibility of root segments (cf. Bauhus and Messier, 1999a). Living spruce and beech roots were identified visually according to root elasticity and root cortex properties: spruce roots are elastic with a relatively thick and irregularly structured brownish cortex, whereas beech roots are less elastic and the red-brown cortex is thin with lines along the longitudinal axis (Schmid, 2002). We studied fine root attributes of spruce and beech with the digital image analysis system “Win-RHIZO V3.10” (Régents Instruments Inc., Canada). The sorted life fine root parts of spruce or beech were placed in a transparent water filled tray (10 × 15 cm) to facilitate root spreading. The system scanned all fine root fragments and the image analyses calculated the architectural traits, root length, and root surface for all fine roots. This method is proved to be reliable; only negligible errors for fine root structure measurements through root overlapping and abutment were found in a test-study by Bauhus and Messier (1999b).

All recorded root fragments were then dried for 48 h at 40°C and weighed in order to measure living fine root biomass (*FRB*, g m^−2^). The reported measurements allowed the calculation of specific root length (*SRL*, m g^−1^) and specific root surface area (SRA) (*SRA*, cm^2^ g^−1^) from the ratio of fine root length (FRL) and root biomass, and fine root surface area and rooting biomass, respectively.

#### Data analyses

The data was included in a relational database running on the open source database system PostgreSQL and analyzed with the software package Statistica 9 (StatSoft, Inc. 2009) and SAS JMP 9 (SAS Institute Inc. 2010). Differences between mean root traits were assessed either by the Kolmogorov-Smirnov-two-sample test, or by the Kruskal-Wallis H test when more than two samples or groups were compared. We used the respective tree BA per hectare of either spruce or beech as a reference unit for the comparison of fine root attributes (*FRA*: biomass, length and surface, cf. Schmid, 2002; Bolte and Villanueva, 2006) of trees or tree groups with different competitive situations (*CT*, Central tree; *COMP*, competitors), and thus variable species abundance above-ground (Equations 1, 2):

(1) $$FRA_{ad,CT}=\frac{FRA_{CT}}{BA_{CT}}$$

(2) $$FRA_{ad,COMP}=\frac{FRA_{COMP}}{{\sum_{1}^{n}BA}_{COMP}}$$

where *FRA* are the life fine root attributes in terms of *FRB* (g m^−2^), *FRL* (m m^−2^), and *RAI* (cm^2^ m^−2^) of a central tree (*CT*) or *n* competitors (*COMP*), respectively. *BA* (m^2^ ha^−1^) is the BA of either spruce or beech, and *FRA*_*ad*_ are fine root attributes adjusted to the same BA (1 m^2^ ha^−1^) of either one central tree or of *n* competitors (*FRB*_*ad*_, kg m^−2^ BA; *FRL*_*ad*_, km m^−2^ BA; *RAI*_*ad*_, m^2^ m^−2^ BA).

The fine root representation of either central tree (*CT*) or competitor (*COMP*) constellations of spruce and beech were further assessed by calculating relative fine root attributes (*rFRA*, Schmid, 2002). The *rFRA* indicator relates the adjusted fine root attributes (*FRA*_*ad*_) in mixed groups with either *CT* or *COMP* constellations to those of pure stand groups (Equation 3). This enables the assessment of under- (<1) or overrepresentation (>1) of beech and spruce below ground growing in different mixed stand constellations under interspecific competition.

(3) $$rFRA=\frac{FRA_{ad,\text{mix}}}{FRA_{ad,\text{pure}}}=\frac{FRA_{ad,CT\text{/COMP}}}{FRA_{ad,\text{pure}}}$$

## Results

### Overall plot means of fine root attributes

Overall core sample means for living fine root attributes show differences between spruce and beech as well as between the two Swedish sites and the German site. At all three sites beech had a 1.3–1.5 fold higher living *FRB* than spruce; an even higher beech-spruce ratio of 2.2–3.3 was found for living *FRL* and a ratio of 1.7–2.4 for living fine root area index (*RAI*, Table 4). Total fine root attributes of beech and spruce are quite comparable for both Swedish sites whereas the German Rehberg site has considerably lower amount of fine roots with less than half of *FRB*, *FRL*, and *RAI*. The lower fine root abundance of trees at the German site is in line with an also lower stand density (see Table 3). Since tree abundance above and below ground is correlated on microsite level (Bolte and Villanueva, 2006; Rewald and Leuschner, 2009; Lang et al., 2010), following detailed comparisons of the different intraspecific group constellations based on all three sites are only valid when using adjusted fine root attributes(cf. Equations 1, 2).

**Table 4:** **Live fine root (*d* ≤ 2 mm) attributes for spruce and beech of all mixed stand plots (*n* = 6 plots per stand)**.

**Stand**	**Rågetaåsen**	**Siggaboda**	**Rehberg**
**LIVE FINE ROOT BIOMASS *FRB*, DRY WEIGHT (g· m^−2^)**			
Spruce	171 ± *212*	192 ± *184*	82 ± *78*
Beech	253 ± *205*	250 ± *211*	106 ± *116*
Total	424 ± *267*	442 ± *211*	188 ± *128*
**LIVE FINE ROOT LENGTH *FRL* (km·m^−2^)**			
Spruce	0.9 ± *1.0*	0.9 ± *0.8*	0.5 ± *0.4*
Beech	3.0 ± *2.5*	2.2 ± *1.7*	1.1 ± *1.1*
Total	3.9 ± *2.4*	3.1 ± *1.6*	1.6 ± *1.1*
**LIVE FINE ROOT AREA INDEX *RAI* (m^2^·m^−2^)**			
Spruce	3.6 ± *4.1*	3.7 ± *3.4*	2.0 ± *1.6*
Beech	8.8 ± *7.2*	7.2 ± *5.7*	3.3 ± *3.3*
Total	12.4 ± *7.1*	10.9 ± *5.2*	5.3 ± *3.3*

*Mean values ± standard deviations of total cores to bedrock depth are displayed; n = 154 samples for Rågetaåsen, n = 148 samples for Rågetaåsen, n = 123 samples for Rehberg*.

### Vertical fine root distribution

**Figure 3:**
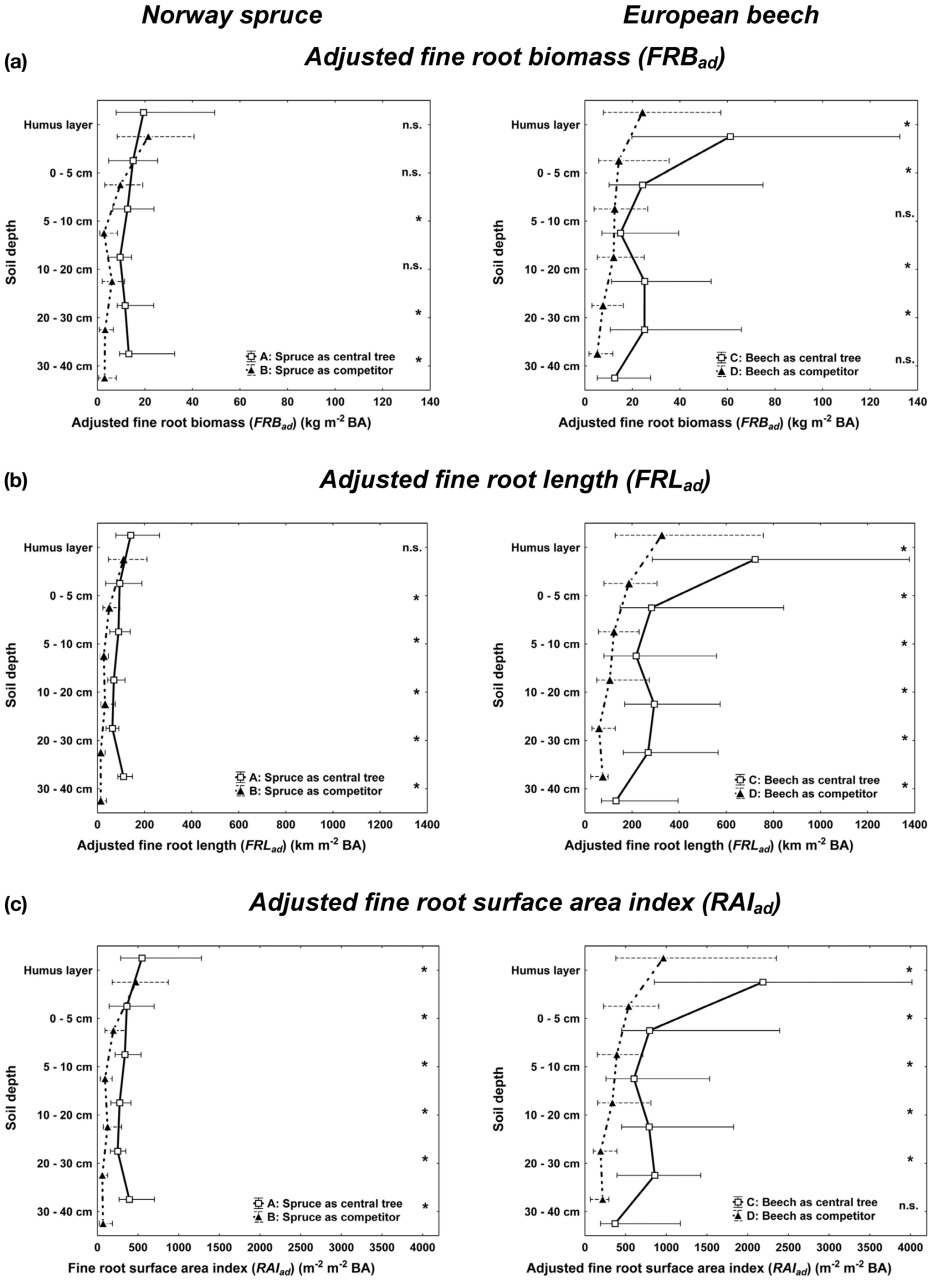
**Vertical distribution of the adjusted fine root parameters for Norway spruce (left; A: spruce as central tree, B: spruce as competitor), and European beech (right; C: beech as central tree, D: beech as competitor).** Whisker plots (median, 25/75 percentile) are shown using root structure data from each sample point adjusted by the BA sums of all trees within the plot area. Stars indicate significant differences between pure and mixed stand values at *p* < 0.05 (Kolmogorov-Smirnov-two-sample test).

For both spruce and beech, central trees (*CT*) attained higher adjusted fine root abundance (*FRB, FRL, RAI*) along the vertical rooting profile than the competitors (*COMP*, Figure 3). This result, however, should be regarded with caution, since it may be biased by comparing the complete rooting area of the central tree with only partial inclusion of the competitors' rooting area. When comparing non-biased allocation shape of fine root abundance among different soil depths (Figure 3), central beech trees (*CT*) exhibited significantly higher fine root abundance in both the humus layer and deeper soil layers compared to beech competitors (*COMP*). This was not the case for spruce with a quite equal allocation of fine root proportion along the profile for both *CT* and *COMP* constellations. A spatial separation of the fine roots of spruce and beech was visible for the deeper soil horizons (10–40 cm) when growing with beech as the central tree (3 **C**, solid line, right) and spruce as competitors (3 **B**, dashed line, left) resulting in high beech and low spruce fine root quantities. This partially vertical stratification was mainly due to high plasticity of beech fine rooting as a central tree. No stratification was visible in the organic layer and top soil where both species reached their maximum abundances.

Total means of specific *SRL* and specific fine root surface area (*SRA*) attained values for mixed beech (*CT* and *COMP*) of *SRL* 12.7 ± 5.5 m g^−1^ and of *SRA* 367.3 ± 125.9 cm^2^ g^−1^ compared to mixed spruce of *SRL* 6.6 ± 3.8 m g^−1^ and of *SRA* 253.6 ± 108.1 cm^2^ g-^1^ (Kolmogorov-Smirnov-two-sample test, *p* < 0.05). However, total *SRL* and *SRA* differences between *CT* and *COMP* constellations within both species were not significant.

**Figure 4:**
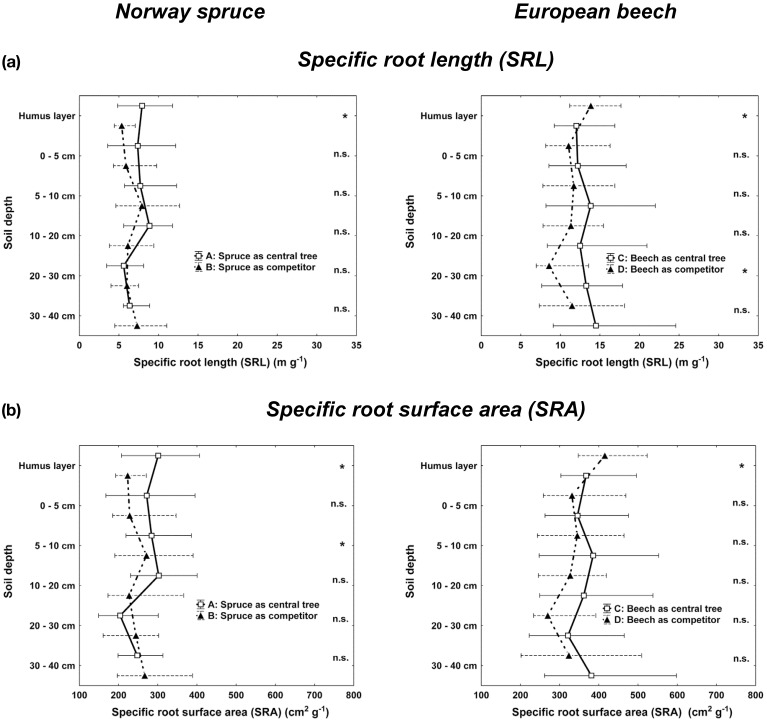
**Vertical distribution of (a) the specific root length (*SRL*) and (b) the specific root surface area (*SRA*) for Norway spruce (left; A: spruce as central tree, B: spruce as competitor), and European beech (right; C: beech as central tree, D: beech as competitor).** Whisker plots (median, 25/75 percentile) are shown. Stars indicate significant differences between pure and mixed stand values at *p* < 0.05 (Kolmogorov-Smirnov-two-sample test).

The vertical profiles of *SRL* and *SRA* support the idea of higher plasticity of beech fine rooting (Figure 4). Whereas beech competitors attained significant higher *SRL* and *SRA* in the humus layer, these values tended to be higher for central beech trees in deeper soil horizons (significant for *SRL* in 20–30 cm depth). For spruce in contrast, we did not find such changes between central spruce trees (*CT*) and its competitors (*COMP*) rooting behavior. However, significant higher SRL and SRA values of *CT* constellations in the humus layer (*SRL, SRA*) and the top soil (*SRA*, 0–5 cm depth) points to higher competitive investments for rooting space sequestration in the upper soil horizons of single central spruce trees (*CT*), whereas single beech trees (*CT*) invested more in deeper soil horizons with less competitive abundance of spruce roots. Comparing beech and spruce as interspecific competitors (*COMP*), one can observe quite similar vertical rooting behavior (both fine root attributes, *FRA* and structural traits, *SRL* and *SRA*), beside the above mentioned fact that the overall level of beech is much higher than that of spruce.

### Fine root representation

For the analyses of fine root representation (*rFRA*, cf. Equation 3) in mixed *CT* and *COMP* constellations (Table 5) we used root samples from pure groups of spruce or beech as reference. This enabled the assessment of the effects of *CT* (A: spruce, D: beech) and *COMP* (B: spruce, D: beech) constellations on interspecific tree species representation compared to the intraspecific one (Table 5, *rFRA*). Two major findings were derived: (1) spruce is underrepresented in fine root abundance in mixed stands (mean *rFRA* < 1) whereas beech is overrepresented (mean *rFRA* > 1); (2) both beech overrepresentation and spruce underrepresentation address *CT* constellations with beech as the central tree (B). This indicates that beech rooting was favoured by an asymmetric interspecific competition of both species when several spruce competitors grew together with a single beech tree. But this was not the case for *CT* constellations with central spruce and beech competitors where we found a quite symmetric interspecific competition. Combining results from the existing mixtures (means of A + D) of central spruce (*CT*) and competing beech (*COMP*) or vice versa (means of B + C) one can assess fine root representation effects of the mixtures compared to either beech or spruce pure groups. It turned out that due to the low fine root representation of spruce no existing mixture reaches the high fine root representation of pure beech. However, both mixture constellations (means A + D, B + C) lead to higher fine root representation compared to the pure spruce plots.

**Table 5:** **Total means of all stands (± *standard deviation*) and relative mixed stand representation compared to pure stand (rFRA, Equation 3) for different adjusted fine root attributes related to central tree (*CT*), competitor (*COMP*) status**.

**Spruce**	**A: CT (*n* = 209)**	**B: COMP (*n* = 169)**	**Pure stand (*n* = 55)**	**rFRA A: *CT*/B: *COMP*/mean**
	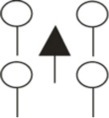	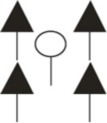	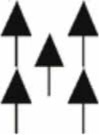	
Fine root biomass (*FRB*_*ad*_) (kg·m^−2^ BA)	61.8 ± *144.6*	44.9 ± 45.8	67.9 ± 72.3	0.91/0.66/0.79
Fine root length (*FRL*_*ad*_) (km·m^−2^ BA)	336.8 ± *725.2*	224.7 ± *194.1*	344.0 ± *356.4*	0.98/0.65/0.82
Fine root area index (*RAI*_*ad*_) (m^−2^·m^−2^ BA)	1356.1 ± 3052.8	922.3 ± 814.9	1382.0 ± 1418.2	0.98/0.67/0.82
**Beech**	**C: CT (*n* = 209)**	**D: COMP (*n* = 169)**	**Pure stand (*n* = 60)**	**rFRA C: *CT*/D: *COMP*/mean**
	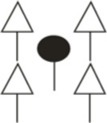	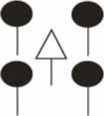	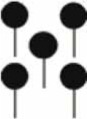	
Fine root biomass (*FRB*_*ad*_) (kg·m^−2^ BA)	164.9 ± *234.7*	131.4 ± *225.4*	135.9 ± *104.9*	1.21/0.97/1.09
Fine root length (*FRL*_*ad*_) (km·m^−2^ BA)	1752.3 ± *2263.1*	1334.3 ± *1887.1*	1495.2 ± *1071.6*	1.17/0.89/1.03
Fine root area index (*RAI*_*ad*_) (m^−2^·m^−2^ BA)	5271.3 ± 7131.0	4166.6 ± 6215.6	4425.8 ± 3156.2	1.19/0.94/1.07

*The samples do not originate from the same distribution (Kruskal-Wallis H-test, p < 0.05)*.

## Discussion

### Overall plot means of fine root attributes

The presented means of the *FRB* (*d* ≤ 2 mm) of the mixed stand plots (and not stand scale) in Sweden (424–442 g m^−2^, Table 4) fit quite well to overall means of an extensive literature study for boreal and temperate forests with 399 ± 239 and 362 ± 182 g m^−2^, respectively (Finér et al., 2011, Table 5, original sampling depth). The lower *FRB* mean for the German Rehberg mixed plots (188 g m^−2^) is related to the remarkably low tree abundance above ground (*BA*, basal area) compared to the Swedish plots. The positive relationship between tree dimension (*BA*) and soil exploration (*FRB)* on microsite and tree level has been demonstrated by Bolte and Villanueva (2006) in mature spruce-beech mixed stands indicating generally stem-centered *FRB* distribution of either spruce or beech with higher values near to large trees of the same species (cf. Rewald and Leuschner, 2009 and also Lang et al., 2010 conspecific beech plots). Values of live *FRL* and *RAI* are found to reflect the resource exploitation ability of plants better by focusing on the sensitive parameter *root length* for soil exploration and *root surface* for resource uptake (cf. Fitter, 2002). Our mixed plot means (Table 4) are in the range of previously reported overall means for boreal and temperate forests of *FRL* 2.6–6.1 and *RAI* 4.6–11.0 (Jackson et al., 1997) except of the lower *FRL* value in Rehberg due to above mentioned reasons.

### Vertical fine root distribution

There is an on-going scientific debate about vertical root system stratification or segregation in mixed stands which is considered to be a major reason of overyielding below ground, i.e., the increase of FRB in mixed vs. pure stands (Schmid, 2002; Meinen et al., 2009a; Lei et al., 2012a; Brassard et al., 2013; Jacob et al., 2013; Smith et al., 2013). There seems to be a general effect of different admixed species and number of mixed species. Root system stratification on stand level was found mainly in (1) two-species systems, mixtures of (2) conifers with broadleaved species as well as of (3) early- or mid-successional tree species with late-successional species. Regarding late-successional, broadleaved beech, root system stratification was observed when admixed in (mainly) two-species stands to Scots pine (*Pinus sylvstris*, MacQueen, 1968; Curt and Prévosto, 2003), Douglas fir (*Pseudotsuga menziesii*, Hendriks and Bianchi, 1995), Norway spruce (Rothe, 1997; Schmid, 2002; Bolte and Villanueva, 2006), Sessile oak (*Quercus petraea*, Büttner and Leuschner, 1994; Leuschner et al., 2001) and common ash (*Fraxinus excelsior*, Rust and Savill, 2000). No evidence for root system stratification was found in multispecies mixtures with beech and other broadleaved tree species (Smith et al., 2013), and including additional late-successional species like winter lime (*Tilia cordata*, Meinen et al., 2009b; Jacob et al., 2013). Our result of only partial root system stratification (*FRB*_*ad*_, *FRL*_*ad*_, and *SRA*_*ad*_) of central beech and competing spruce in deeper soil horizons (Figure 3) draws the attention to the question *where* and under *which conditions* root system stratification occurs. Stratification may only occur in stand areas where the root systems of the competing tree species considerably overlap. In particular beech fine rooting are found not to be “territorial” (Lang et al., 2010) and may exploring soils near to an interspecific competitor (e.g., Büttner and Leuschner, 1994; Rewald and Leuschner, 2009). However, we found in our design several sample points with no considerable fine root abundance of either spruce or beech, and consequently no root system stratification. In a previous study (Bolte and Villanueva, 2006) we focussed our root sampling on the overlapping rooting zones of spruce and beech between conspecific groups of both species. There, we found strong root system stratification. This indicates that sampling design and specific location of root sampling affect results on vertical rooting and stratification, and limits the generalization options for entire stands. Thus, rooting information taken from specific structural sub-strata or from location of even unknown structural stratum should be treated with care when using them for general statements on a stand level. Another condition for root system stratification is the availability of (non- or less-occupied) rooting space in deeper soil horizons. Apart from chemical restrictions like oxygen deficiency (e.g., Pezeshki and Santos, 1998) as well as soil acidification and related mobilization of root-toxic aluminium ions (Cronan and Grigal, 1995), physical restrictions and varied conditions for the exploration of deeper soil horizons like a massive bedrock layer or boulder occurrence may limit fine root system stratification. An important condition for active root system stratification by shifting fine root abundance to lower soil layers is a successful change of rooting behavior, and thus acclimation, in terms of “optimality” (“Optimality theory,” Bloom et al., 1985; Parker and Maynard Smith, 1990; Eissenstat and Yanai, 1997). There has to be a positive effect in the “cost/gain ratio” to invest resources to change its rooting, and the selected plasticity (Grime et al., 1986; De Kron and Mommer, 2006). Where a large resource availability gradient from high availability in the top soil to a lower one in the deeper soil exists there is little “gain” to change rooting behavior. The same applies if the favourable soil space is already occupied by tree species with similar rooting plasticity and foraging strategy that could be explained by similar plant strategy type (Grime, 1979) and successional status (Bolte and Villanueva, 2006; Meinen et al., 2009a; Jacob et al., 2013). In our study, the different strategy of mid-successional conifer spruce with more conservative rooting and late-successional broadleaved beech with plastic rooting points to root system stratification in the overlapping zone. However, physical and chemical rooting restrictions in the boulder-rich soils with nutrition status of the mineral soil seemed to counteract a complete stratification (with beech as central tree) and to stimulate beech to compete intensively with spruce in the humus layer and uppermost soil horizons.

*SRL* of living fine roots can explain economic aspects of fine root system morphology (Ostonen et al., 2007), as does specific *SRA* (*SRA*, Bolte and Villanueva, 2006). Our *SRL* means for mixed spruce (6.6 m g^−1^) and mixed beech (12.7 m g^−1^) lie within the lower part of the range for spruce and beech (*d* < 2 mm) of 4.5–26 and 5.7–31.5 m g^−1^, respectively, reported by the extensive meta-analyses of Ostonen et al. (2007). The *SRL* and *SRA* means (spruce 253.6 cm^2^ g^−1^, beech 367.3 cm^2^ g^−1^) are similar to those reported for mixed spruce by Bolte and Villanueva (2006) but lower than those for mixed beech. Along the vertical profile (Figure 4), *SRL* for mixed beech and spruce are lower than those found for young mixed stands by Lei et al. (2012b). The higher *SRL* and *SRA* values for beech compared to spruce correspond to ideas of basic differences of deciduous angiosperms and conifers, latter having thicker roots and thus lower *SRL* and *SRA* (Bauhus and Messier, 1999a; Lei et al., 2012b).

In line with above mentioned “optimality theory” and economic “cost/gain evaluations,” *SRL* is used as an indicator parameter for space sequestration efficiency (*SSE*, Grams et al., 2002): fine root systems with a higher *SRL* are supposed to sequester rooting space with lower “carbon” costs (Ostonen et al., 2007). In this respect, beech is more efficient to explore root space which is one explanation for higher rooting plasticity of beech compared to spruce. However, higher soil exploration efficiency (*SSE*) of beech does not mean that resource exploitation is more efficient compared to spruce (Lei et al., 2012b). In contrast to other studies which report a decrease of *SRL* with increasing soil depth (Bauhus and Messier, 1999a; Lei et al., 2012b), mixed central beech (*CT*) increased *SRL* in deeper soil layers. This indicates additional soil exploration activities at low carbon costs in soil depths from 20 cm downwards fitting well to the findings of changed root system partitioning favouring deeper soil horizons for increasing complementary resource exploitation and “underground niche separation” (Parrish and Bazzaz, 1976).

### Fine root representation

The relative fine root representation is a measure to compare mixed species abundance (*FRB, FRL, RAI*) adjusted to the same unit of above ground performance (1 m^2^ BA per hectare) with adjusted values for monospecies plots (cf. Schmid, 2002; Bolte and Villanueva, 2006). The underrepresentation of mixed spruce (Table 5, mean *rFRA*) is in line with other studies reporting an over-proportional reduction of fine root abundance in beech mixtures with spruce (Schmid and Kazda, 2002; Bolte and Villanueva, 2006), oak (Büttner and Leuschner, 1994; Leuschner et al., 2001; Rewald and Leuschner, 2009), common ash (Rust and Savill, 2000), or Douglas fir (Hendriks and Bianchi, 1995). This finding is contrasted by a slight overrepresentation of beech fine root abundance indicating an asymmetric interspecific competition of spruce and beech that was also found by Schmid (2002) but not by Bolte and Villanueva (2006). Previously reported ideas of a low belowground competitive ability of spruce compared to beech in mixed mature stands (Schmid, 2002; Bolte and Villanueva, 2006) are supported by this study. In particular, spruce competitors (*COMP*) surrounding a single beech tree (*CT*) have a low fine root representation possibly reflecting intensive above ground competition between central beech and spruce competitors leading to increased biomass partitioning toward aboveground tree compartments. Beech fine root representation on the other hand is favored by interspecific competition with spruce. The found morphological belowground plasticity (variation of rooting depth in *CT* constellation and of *SRL/SRA*) reflects the high crown plasticity (Dieler and Pretzsch, 2013) and the overall “foraging” ability of beech in relation to different growth resources. According to the coherence (beech) or dissimilarity (spruce) of competitive response above and below ground, the linkage of competition assessments above and below ground are of increasing interest.

## Concluding remarks

The results of the presented study exhibited an only partially vertical root system stratification in the subsoil in the three spruce-beech old-growth stands and depended on specific mixture constellation: beech central tree with spruce competitors. In this mixture constellation, beech was able to raise *SRL* and with this soil exploration efficiency in the subsoils while increasing root biomass partitioning toward deeper soil layers. Moreover, asymmetric below-ground competition was observed favoring beech toward spruce in a mixed constellation with central beech. We conclude that beech fine rooting is facilitated in the presence of spruce by lowering competitive pressure compared to intraspecific competition whereas the competitive pressure for spruce is increased by beech admixture. This is most obvious when central beech trees are admixed with spruce competitors. Our findings underline the need of spatially differentiated approaches to assess interspecific competition below ground. Since tree competition is a process that affects tree at an individual scale, stand scale analyses should be complemented by single tree approaches, above and below ground. In line with the recent development of crown competition assessments and stand simulation methods (e.g., Pretzsch et al., 2002; Nagel et al., 2006), single-tree approaches and simulations of below-ground competition are required to focus rather on microsites populated by individual specimens as the basic spatial study area.

### Conflict of interest statement

The authors declare that the research was conducted in the absence of any commercial or financial relationships that could be construed as a potential conflict of interest.
